# The gut microbiome in dogs with congestive heart failure: a pilot study

**DOI:** 10.1038/s41598-020-70826-0

**Published:** 2020-08-13

**Authors:** Joonbum Seo, Linda Matthewman, Dong Xia, Jenny Wilshaw, Yu-Mei Chang, David J. Connolly

**Affiliations:** 1 Clinical Science and Services, Royal Veterinary College, North Mymms, England, UK; 2 Department of Pathology and Population Sciences, Royal Veterinary College, North Mymms, England, UK; 3 Research Support Office, Royal Veterinary College, North Mymms, England, UK

**Keywords:** Cardiovascular biology, Gastrointestinal models

## Abstract

Compromised gut health and dysbiosis in people with heart failure has received a great deal of attention over the last decade. Whether dogs with heart failure have a similar dysbiosis pattern to what is described in people is currently unknown. We hypothesised that dogs with congestive heart failure have quantifiable dysbiosis compared to healthy dogs that are similar in sex and age. A total of 50 dogs (15 healthy dogs and 35 dogs with congestive heart failure) were prospectively recruited, and their faecal gut microbiome was assessed using 16S rRNA sequencing (Illumina MiSeq platform). There was no significant change in the microbial diversity and richness in dogs with congestive heart failure. However, there was an increase in abundance of Proteobacteria in the congestive heart failure group (*p* = 0.014), particularly due to an increase in the family *Enterobacteriaceae* (*p* = 0.002) and *Escherichia coli* (*p* = 0.033). We conclude that dogs with congestive heart failure have dysbiosis, and we show additional trends in our data suggesting that dogs may have a similar pattern to that described in people. The results of this study provide useful preliminary information and raise the possibility that dogs represent a clinically relevant animal model of dysbiosis in people with heart failure.

## Introduction

The gut microbiota is a consortium of microorganisms in the digestive tract^1^. In addition to contributing to digestion, these organisms function as an endocrine organ through the production of bioactive metabolites and modulate the immune system through the translocation of immunomodulatory bacterial products across the intestinal wall^1^. In people, alteration in the microbiota, also known as dysbiosis, is recognised in diseases including chronic kidney disease, inflammatory bowel disease, heart failure and obesity, suggesting involvement of the microbiota in their pathophysiology and progression^1–8^. Similar changes in the microbiota have been identified for some of these diseases in veterinary medicine, but not for heart failure to date^9–13^.

The contribution of the gut microbiota in the development and maintenance of heart failure known as the “gut-hypothesis” details impaired gut health in heart failure patients, resulting in dysbiosis and bacterial translocation across the oedematous mucosal layer^14–16^. This results in immune-stimulation via endotoxins, contributing to a chronic inflammatory state, malnutrition and cachexia. While this has been the focus for evaluation of novel therapies in people with advanced heart failure, no such research has been described in veterinary medicine^1,16,17^. Congestive heart failure (CHF) is a common cardiac presentation in dogs, with typical signs comprising severe respiratory distress, due to pulmonary oedema or pleural effusion or abdominal distension due to ascites. Medical management is successful in many cases, but dogs with CHF, particularly those with right sided congestive heart failure (RCHF), are often euthanised due to progressive inappetence and severe cachexia, even when their heart failure is adequately managed^18^. Reduced appetite and cachexia are also recognized in dogs with left sided congestive heart failure (LCHF), due to acquired mitral valve disease, and are independently associated with outcome^19,20^. Evaluation of the gut microbiota in these dogs could enhance our understanding of gut health in heart failure and the subsequent deleterious pro-inflammatory systemic sequelae, such as cardiac cachexia. If dogs show a similar dysbiosis pattern to people, they could potentially serve as a clinically relevant animal model for people with heart failure.

## Results

### Study population

A total of 50 dogs were enrolled, of which 15 were healthy (control) and 35 had CHF. Seventeen dogs in the CHF group were living with other dogs in the same household. Eight of these cohabiting dogs were included in the control group. The CHF group comprised 16 LCHF, 15 RCHF, and 4 biventricular congestive heart failure (BiCHF) dogs. The causes of CHF were myxomatous mitral valve degeneration (n = 19), dilated cardiomyopathy (n = 13), congenital heart disease affecting the tricuspid and pulmonic valves (n = 2), and pulmonary hypertension (n = 1). There was no difference in age, body weight or sex between the control and CHF groups. However, the CHF group had a lower body condition score (BCS) (*p* = 0.005), muscle condition score (MCS) (*p* < 0.001) and appetite (*p* < 0.001) than the control group. All healthy dogs and most CHF dogs (96.0%) were eating commercial dog food as their primary diet, however, more owners were supplementing their dogs with human foods in the CHF group (5/15 control dogs vs 23/35 CHF dogs, *p* = 0.061). These human foods consisted mainly of what the owner was eating on the day and were mostly protein based with no added salt (i.e. cooked chicken or white fish). Two dogs with CHF were exclusively eating human foods (cooked lamb and pasta for one dog, cooked chicken and vegetables for the other). Further demographic data is listed in Table 1. Additional information on breed and diet for each CHF subgroup (LCHF, RCHF and BiCHF) is listed in Supplementary Table S1 online.

**Table 1:** Demographic characteristics of the control and congestive heart failure (CHF) groups.

	Control (n = 15)	CHF (n = 35)	*p* value
**Age (years)**	8.0 [6.9–10.0]	10.2 [7.9–10.9]	0.141
**Sex**			0.462
Female (%)	7 (46.7%)	10 (28.6%)	
Neutered: entire	7:0	10:0	
Male (%)	8 (53.3%)	25 (71.4%)	
Neutered: entire	5:3	15:10	
**Breeds (%)**			0.106
Small breeds	4 (26.7%)	18 (51.4%)	
Large breeds	11 (73.3%)	17 (48.6%)	
**Body weight (kg)**	20.3 [12.1–33.0]	12.0 [7.3–42.5]	0.352
**BCS (/9)**			**0.005**
1/9	0	1 (2.9%)	
2/9	0	4 (11.4%)	
3/9	0	8 (22.9%)	
4/9	3 (20.0%)	4 (40.0%)	
5/9	6 (40.0%)	6 (17.1%)	
6/9	6 (40.0%)	2 (5.7%)	
**Muscle condition**^**a**^			**< 0.001**
Normal	15 (100%)	8 (22.8%)	
Mild	0	11 (31.4%)	
Moderate	0	9 (25.7%)	
Severe	0	6 (17.1%)	
**Diet**			
Dog food	15 (100%)	33 (94.3%)	1.000
Human food	5 (33.3%)	23 (65.7%)	0.061
**Appetite**			**< 0.001**
Normal	15 (100%)	13 (37.1%)	
Reduced	0	22 (62.9%)	
**Duration of CHF (weeks)**	–	6.0 [3.0–28.0]	–
**Medication**			
Diuretic dose (mg/kg/day)	–	4.4 [3.4–7.1]	–
Furosemide	0	28 (80.0%)	–
Torsemide	0	4 (11.4%)	–
Pimobendan	0	33 (94.3%)	–
Benazepril	0	25 (71.4%)	–
Spironolactone	0	23 (65.7%)	–
Fish oil	0	4 (11.4%)	–
Diltiazem/digoxin	0	9 (25.7%)	–

^a^One dog in the CHF group was missing a muscle condition score. The results are displayed as median [25th, 75th percentiles] as they were non-normally distributed. Statistical significance (*p* < 0.05) is marked bold.

### Alpha and beta diversity

**Figure 1:**
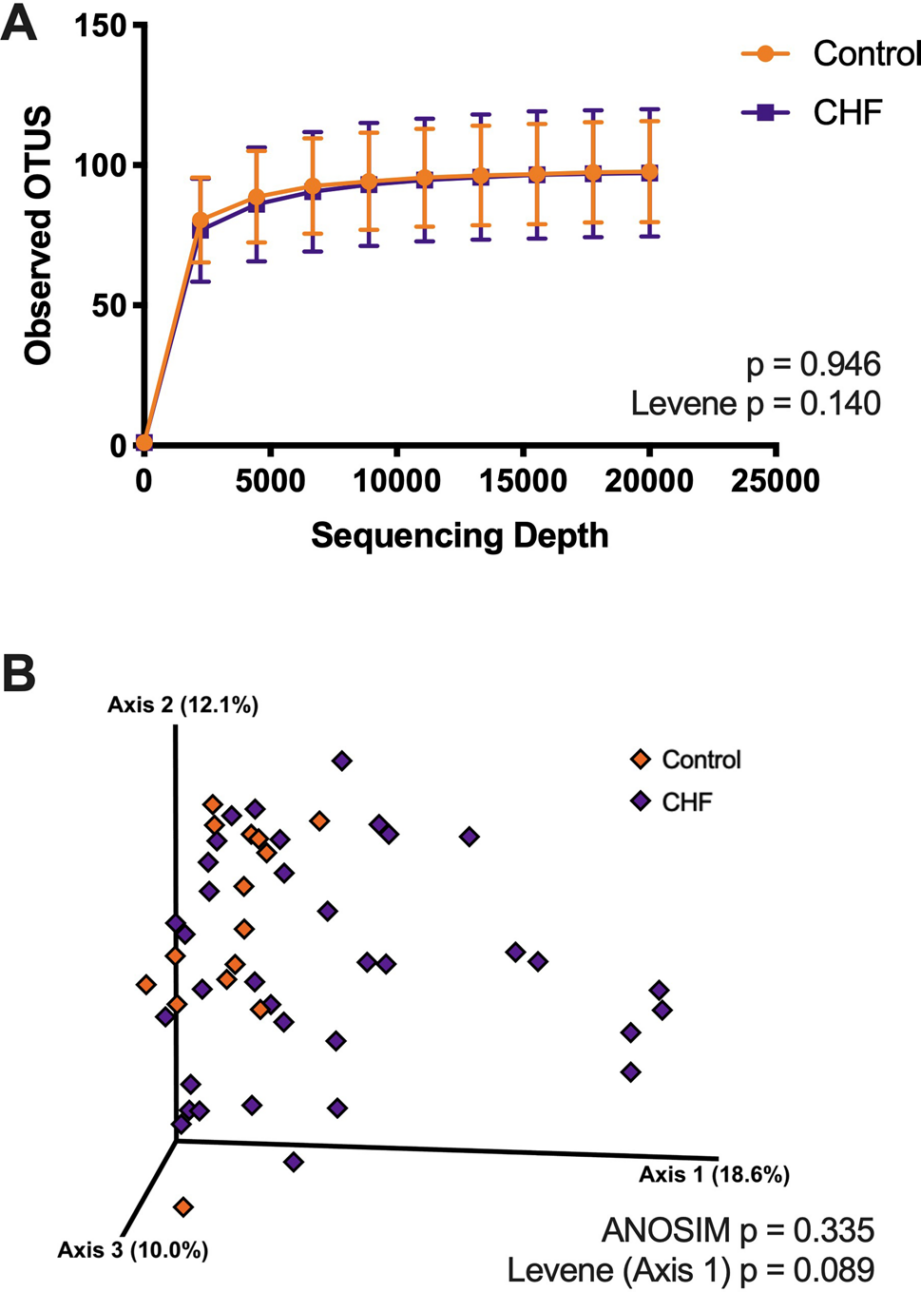
Comparison of alpha (**A**) and beta diversity (**B**) between the control and congestive heart failure (CHF) groups. The line in the alpha rarefaction curves (**A**) marks the mean and the error bars mark the standard deviation. The principle coordinate analysis plot shows the results of Bray–Curtis dissimilarity analysis (**B**). There was no statistical significance between the two groups in both analyses.

There were 28,374,445 reads generated by the Illumina sequencing of the 50 samples submitted. The assessment of alpha diversity is summarised in Table 2. There was no significant difference in the alpha diversity between the control versus CHF groups when assessed using operational taxonomic unit, Shannon diversity index or Faith’s phylogenetic diversity (Fig. 1). Beta diversity, analysed based on Bray–Curtis distances, also showed no significant differences between the control versus CHF groups (ANOSIM; R = 0.014, *p* = 0.335). However, a visual assessment of the principle coordinate analysis plot and Levene’s test suggested a trend towards having a greater variation in the results of beta diversity in dogs with CHF (Levene’s test F(1,48) = 3.020, *p* = 0.089). Additional analyses of the subgroups of CHF and CHF dogs living with or without other CHF or healthy dogs also showed no difference in alpha and beta diversity between the groups (see Supplementary Fig. S1 online).

**Table 2:** The alpha diversity results from the 16S rRNA sequencing.

Alpha diversity	Control (n = 15)	CHF (n = 35)	*p* value
OTU	97.7 (± 18.0)	97.2 (± 22.7)	0.946
Shannon index	4.3 (± 0.6)	4.2 (± 0.6)	0.575
Faith’s PD	10.7 (± 1.8)	11.1 (± 2.0)	0.481

The results are displayed as mean (± s.d.) as they were normally distributed.

*CHF* congestive heart failure, *OTU* operational taxonomic units, *Faith’s PD* Faith’s phylogenetic diversity.

### Taxa analysis

**Figure 2:**
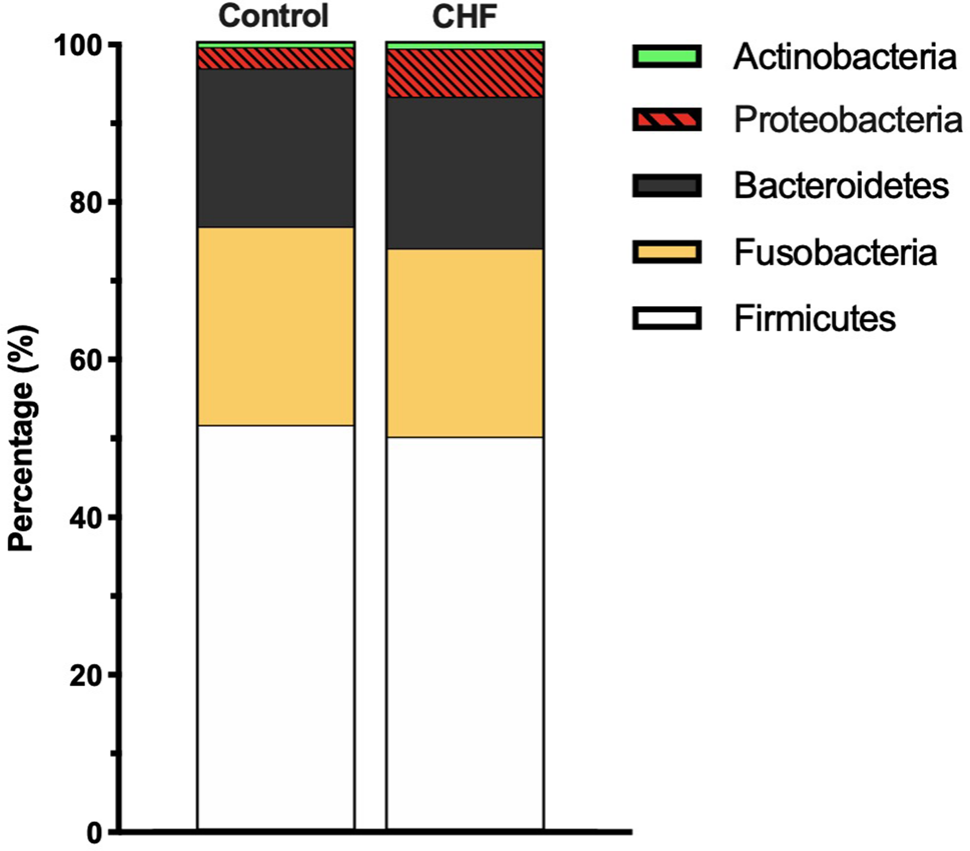
Stacked bar graphs showing the median percentage abundance of gut microbiota at the Phylum level.

**Figure 3:**
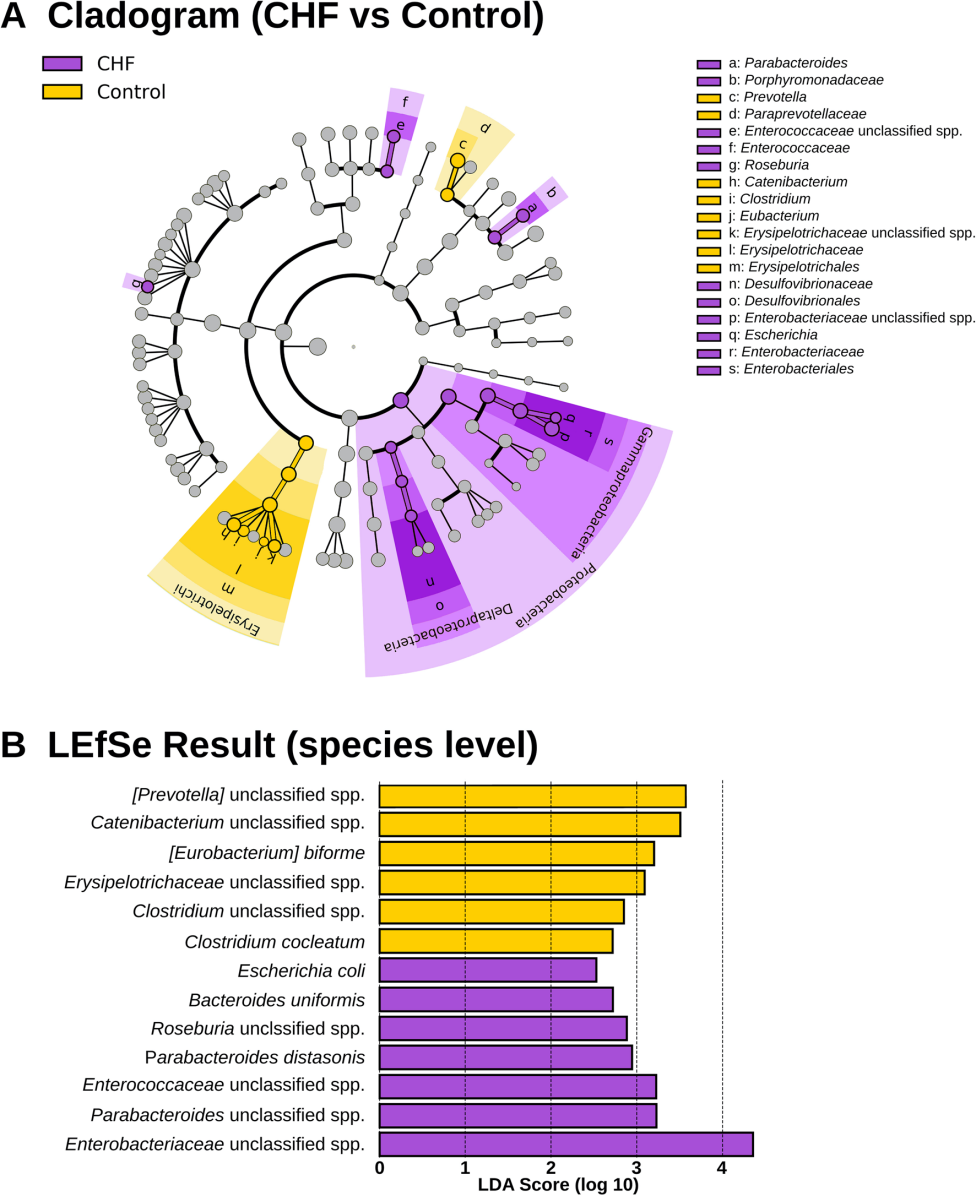
Results of linear discriminant analysis effect size (LEfSe). The cladogram (**A**) shows differentially abundant bacterial taxa from the phylum to the species level. The LEfSe result at the species level is further displayed in a plot (**B**). Only LEfSe scores > 2 are shown.

**Figure 4:**
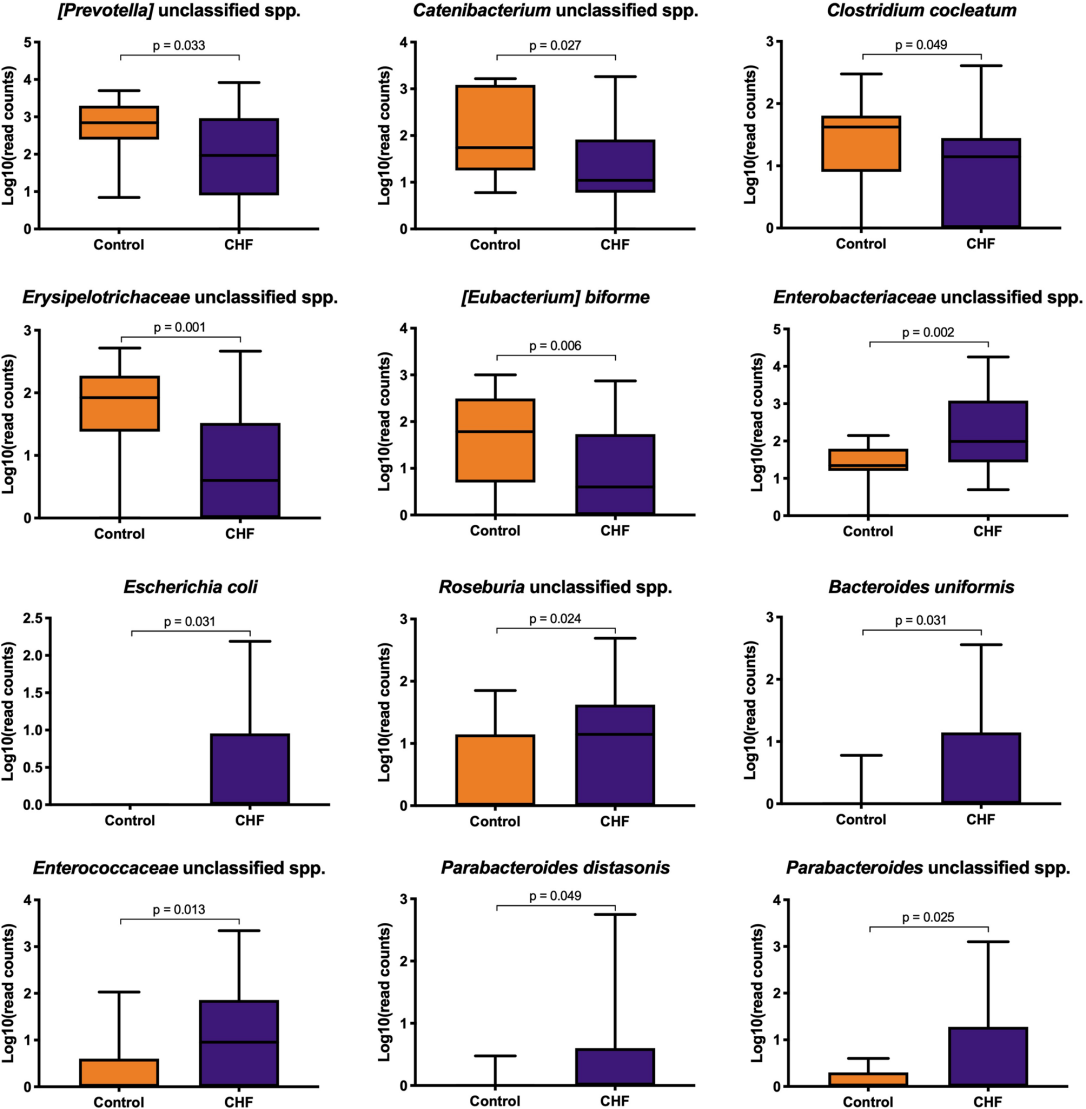
Box plots showing the results of 16S rRNA sequencing of differentially abundant bacterial species. The box shows the respective 25th and 75th percentiles, the line within the box represented median value, and the whiskers show the lowest and highest data points still within 1.5 times the interquartile range of the respective lower and upper quartiles.

Taxa analysis showed 5 main Phyla: Firmicutes, Fusobacteria, Bacteroidetes, Proteobacteria, and Actinobacteria. There was a higher abundance of Proteobacteria in CHF dogs compared to the control dogs (*p* = 0.014; Table 3; Fig. 2). Linear discriminant analysis to the species level showed an increase in the abundance of *Escherichia coli* (*p* = 0.031) and an unclassified species of *Enterobacteriaceae* (*p* = 0.002) within the Proteobacteria of the CHF group. Other species with a relative increase in abundance in the CHF group were an unclassified species of *Roseburia* (*p* = 0.024), an unclassified species of *Enterococcaceae* (*p* = 0.013), *Parabacteroides distasonis* (*p* = 0.049), an unclassified species of *Parabacteroides* (*p* = 0.025), and *Bacteroides uniformis* (*p* = 0.031). In contrast, healthy dogs had an increased abundance of 5 bacterial species, which were: an unclassified species of *[Prevotella]* (*p* = 0.033), an unclassified species of *Catenibacterium* (*p* = 0.027), *Clostridium cocleatum* (*p* = 0.049), an unclassified species of *Erysipelotrichaceae* (*p* = 0.001) and *[Eubacterium] biforme* (*p* = 0.006). Further results are displayed in Figs. 3, 4, and Supplementary Table S2 online.

**Table 3:** Taxa analysis at Phylum level.

Phylum	Control (n = 15)	CHF (n = 35)	*p* value
Firmicutes (%)	46.8 [36.7–57.0]	47.3 [31.3–62.4]	0.907
Fusobacteria (%)	25.8 [19.6–36.6]	20.6 [9.9–33.4]	0.153
Bacteroides (%)	17.7 [10.1–25.2]	17.7 [6.7–27.6]	0.975
Proteobacteria (%)	2.6 [1.0–4.9]	6.6 [2.4–11.7]	**0.014**
Actinobacteria (%)	0.6 [0.2–1.7]	0.8 [0.2–1.6]	0.759

The median percentage abundance [25th, 75th percetiles] is displayed for the control and congestive heart failure (CHF) groups. Statistical significance (*p* < 0.05) is marked bold.

Although there was a statistical difference at the Phyla level between the control and CHF groups, comparing the subgroups of CHF did not show a statistical significance (Proteobacteria Kruskal–Wallis *p* = 0.104; see Supplementary Table S3 online). This suggested that the sample size for comparing multiple subgroups of CHF was underpowered. Therefore, further taxa analysis for subgroups of CHF was not performed.

Results of linear models showed that only 9 species were significantly associated with clinicopathologic data. For each of these 9 species, only one variable remained significant following multivariable analysis. These were: the presence of CHF for the unclassified species of *Enterococcaceae* (*p* = 0.019), *E. coli* (*p* = 0.023), *[Eubacterium] biforme* (*p* = 0.035), and the unclassified species of *Erysipelotrichaceae* (*p* = 0.040); total daily diuretic dose for the unclassified species of *Enterobacteriaceae* (*p* = 0.002); MCS for the unclassified species of *Roseburia* (*p* = 0.017), and the unclassified species of *Parabacteroides* (*p* = 0.025); giving human food for the unclassified species of *Catenibacterium* (*p* = 0.029); and finally, living with another dog (in this case, a control dog) for the unclassified species of [*Prevotella*] (*p* = 0.031).

## Discussion

In this pilot study we compared the gut microbiome in dogs with CHF to healthy control dogs that were similar in age, sex, size and diet. Our results showed that quantifiable dysbiosis occurs in dogs with CHF, with the most pronounced finding being an increase in the abundance of Proteobacteria, particularly due to an increase in *E. coli* and an unclassified species of *Enterobacteriaceae*. While our pilot study design and the small sample size precluded us from detecting less pronounced effects of CHF on the gut microbiota and from performing subgroup analysis with an adequate statistical power, visual and statistical assessment of our data still suggested a greater variation in beta diversity, and a decrease in the abundance of Firmicutes species with butyrate producing potential, which is the more consistent dysbiosis pattern described in people with heart failure^21–25^. A larger sample size is needed to confirm our findings in dogs, but our findings provide valuable preliminary information and support the concept that dogs may represent a clinically relevant animal model for dysbiosis in people with heart failure.

A recent canine study suggested that gut health is impaired in CHF by demonstrating greater concentrations of trimethylamine N-oxide (TMAO) in dogs with CHF^26–28^. In a normal gut, certain gut microbes use dietary nutrients to produce trimethylamine, which is the precursor of TMAO. Bacteria capable of trimethylamine production include Gammaproteobacteria (*E. coli*, *Citrobacter* spp., *Klebsiella pneumoniae*, *Providencia* spp., and Shigella), Betaproteobacteria (*Achromobacter* spp.), Firmicutes (*Sporosarcina* spp.), and Actinobacteria^27,28^. Actinobacteria and *E. coli,* were detected in our study, with the latter only found in the dogs with CHF. The increased TMAO concentrations in CHF^26^, could therefore have been associated with an increased abundance of *E. coli*.

An increase in the number of *E. coli* in dogs with CHF in this study is also consistent with a previous culturomic study in human patients with heart disease^4^ and may correspond with a vulnerable state of the gastrointestinal tract common to both species^29^. Similarly, the abundance of *Enterococcaceae* was increased in dogs with CHF. Metabolic studies have shown that certain genera of *Enterococcaceae*, *E. coli* and other related species may be more competitive in conditions of oxidative stress due to their ability to compensate with glutathione production^30^. This suggests that these bacteria are opportunistic and while some strains of *E. coli* are benign, some contain features that are compatible with pathobionts^30^. *Enterococcaceae* and pathological strains of *E. coli* could induce inflammation, which may contribute to the inappetence, malnutrition and cachexia seen in dogs with CHF^29,31^.

In this study we have chosen 2 weeks to define chronic CHF. That said, CHF in dogs is generally considered a clinical manifestation of a chronic underlying heart condition since dogs tend to hide their symptoms and mild CHF is often missed by owners until it progresses to a point when the dog displays obvious signs such as tachpnoea and marked exercise intolerance. Although we have defined chronic CHF as 2 weeks, it is very likely that these dogs had mild but unobserved signs of CHF for a significantly longer time period, more aligned with the clinical entity of chronic heart failure in human medicine.

One of the confounding variables assessed in this study was the effect of living with another dog. The presence of a dog in the house influences the gut microbiota composition in people^32^ and a similar effect was seen in dogs themselves, as they groom each other and sometimes exhibit coprophagy^33^. These behaviours could introduce different bacterial species that would otherwise not be present, leading to changes in the gut microbiota. When the CHF group was subclassified, a trend in increasing alpha- and beta diversity was seen in those living with a healthy control dog, although this was not statistically significant, probably due to the small sample size (see Supplementary Fig. S1 online). In our multivariable analysis, the greater abundance of the unclassified species of [*Prevotella*] in the healthy dogs was independently associated with living with CHF dogs. Alteration in [*Prevotella*] spp. has previously been observed in rhesus monkeys that underwent a change in social structure^34^. This, or a chance effect from a small sample size, may explain the contradictory finding regarding the [*Prevotella*] abundance between our pilot study and the largest study in people on heart failure and the gut microbiome where the [*Prevotella*] abundance was increased with heart failure^23^.

A proportion of dogs will lose their appetite with the development of CHF and when this occurs, the owners are often advised to feed different types of food in an attempt to meet the daily caloric requirement and minimise the effects of cardiac cachexia. Many owners share some of their meal with their dogs, and in line with veterinary advice, the shared foods are mostly protein based with no added salt (i.e., a portion of cooked chicken). This feeding behaviour has previously been described in a questionnaire-based study in client owned dogs with CHF^35^. In our study, only two dogs with CHF were found to be primarily eating human foods, while the remaining dogs with CHF were still eating commercial dog food as their primary diet, with occasional supplementation using human food. We have accounted for some of this variation in diet by also keeping the diet heterogenous in the control group and by performing a multivariable analysis with the human food as one of the explanatory variables. Whilst this does not completely remove the subtle variation of nutrition in the dogs with CHF, only one bacterial species was found to be influenced by dogs eating human food. This was the unclassified species of *Catenibacterium* (family *Erysipelotrichaceae*), which was decreased in abundance in dogs with CHF. The clinical significance of the loss of *Erysipelotrichaceae* with CHF is unknown, although a depletion of core microbiota such as this, is a feature of dysbiosis in many diseases including heart failure, inflammatory bowel disease and diabetes^13,22,31,36^.

Our study has a number of limitations, mostly as a result of the inherent nature of a pilot study. As a result of the sample size we were only able to detect the most pronounced effect of CHF on the composition of the gut microbiome. Despite this, our data did suggest that the pattern of dysbiosis we observed appears to have similarities to that observed in people with heart failure. Comparing multiple subgroups of CHF was not possible due to the lack of statistical power, and the results of the alpha- and beta diversity and taxa analyses therefore been shared as supplementary files. Similarly, we have found that some of the bacterial species that were significantly associated with the presence or absence of CHF in the linear discriminant analysis and Mann–Whitney U test, were not significantly associated with any of the clinicopathological variables assessed, including the presence or absence of CHF, in a linear model. These were *Clostridium cocleatum, Parabacteroides distasonis,* and *Bacteroides uniformis*. However, the results for both the linear model and taxa analysis of multiple subgroups of CHF suggest that a larger sample size is required to rule out type II error. Another limitation in this study is the assumption of good health in the control dogs. The dogs were deemed healthy on the basis of the history and a careful general and cardiac physical examination. Echocardiography was performed when there was a low-grade heart murmur or breed predisposition, to rule out an underlying cardiac disease. However, health screening blood tests were not performed in any dogs in the control group and whether some of the dogs had an early or pre-clinical systemic disease, such as chronic kidney disease, is unknown.

Overall, we have found that dogs with CHF have quantifiable dysbiosis with an increase in abundance of Proteobacteria (*Enterobacteriaceae* and *E. coli*). The results of this pilot study provide useful preliminary information and raise the possibility that dogs may represent a useful animal model of dysbiosis in people with heart failure, opening up the possibility of translatable clinical trials.

## Materials and methods

### Study design

This was a prospective, case-controlled pilot study, conducted at the Queen Mother Hospital for Animals (North Mymms, Royal Veterinary College) and a research clinic held at a first opinion practice by a diploma holding veterinary cardiologist (Camden, Royal Veterinary College). Ethics approval was provided by the Royal Veterinary College (M20160107).

### Animals

Client owned dogs with either LCHF, RCHF or BiCHF of at least 2 weeks duration were recruited. The 2 weeks of duration was chosen for ‘chronic’ CHF. All dogs in the CHF group underwent echocardiography by board certified or eligible veterinary cardiologists. Left congestive heart failure was defined as the presence of left heart disease with left atrial enlargement, pulmonary oedema detected on thoracic ultrasound or radiographs, and tachypnoea that resolved with furosemide. Right congestive heart failure was defined as the presence of right heart disease, right atrial enlargement and ascites that resolved or improved with furosemide. Lastly, BiCHF was defined when both criteria of LCHF and RCHF were met. Exclusion criteria were the use of an antibiotic or probiotic within 6 months prior to the sample collection, and any suspicion of an underlying enteropathy on the basis of history and review of available blood results.

Healthy dogs were enrolled into the control group while keeping the age and sex similar between the groups. Health was determined based on history and physical examination. Echocardiography was performed for breeds that were susceptible to dilated cardiomyopathy phenotype to rule out observable cardiac disease. When available, a healthy dog cohabiting with a dog in the CHF group was recruited as a control dog. The remaining dogs were owned by the hospital staff where the study was performed.

### Data collection

Data collection included the signalment, diet, oral supplements, medications, appetite perceived by the owner (normal or reduced), number of dogs cohabiting with another dog, body weight, BCS^37^ and MCS^37^. Cohabiting dogs were defined as those spending at least 80% of the day, for at least 6 months with the enrolled dog prior to the time of data collection^38^. When dogs were receiving torsemide, the equivalent dose of furosemide was calculated by multiplying the torsemide dose by 20 for the purposes of statistical analysis^39^.

### Faecal collection and DNA analysis

Samples collected following spontaneous defecation or by rectal extraction were promptly stored at − 80 °C until the faecal DNA was extracted as a batch, using the QIAGEN DNeasy PowerSoil Kit (previously known as MoBio PowerSoil DNA Isolation Kit, QIAGEN), which has been validated for this work^40^. The eluted DNA sample was then shipped to an external laboratory (Molecular Research LP, Mr DNA, Shallowater, TX, https://www.mrdnalab.com) for 16S rRNA sequencing (Illumina MiSeq platform) as previously described^41^. The QIIME 2 pipeline^10^ was used to analyse the faecal microbial communities, measuring the species present and their richness and diversity (α and β diversity), in the apparently healthy and affected dogs.

### Statistical analysis

R (Version 3.6.2, R Foundation for Statistical Computing, Vienna, Austria), SPSS (Version 26.0.0.0, IBM Company, Chicago, IL), and Galaxy Hutlab server (https://huttenhower.sph.harvard.edu/galaxy) were used for statistical analysis. GraphPad Prism 7 (GraphPad Software, San Diego, Ca) was used for production of graphs. Normality was assessed using Shapiro–Wilk tests and histograms. Normally distributed data were summarised as mean (± s.d.) and non-normally distributed data were summarised as median [25th, 75th percentiles]. Depending on the distribution of data, continuous variables were compared using a student *t* test or Mann–Whitney test for 2 groups, and a one-way ANOVA or Kruskal–Wallis test for > 2 groups. Post-hoc analysis was performed using Tukey’s HSD test. Categorical variables were compared using Chi-squared tests. Alpha diversity was assessed by comparing operational taxonomic units, Faith’s phylogenetic diversity and Shannon Index between the groups using a student *t* test or Mann–Whitney test. Analysis of similarities (ANOSIM) and principal coordinate analysis were performed to assess the difference in beta diversity. For comparison of both alpha- and beta-diversity, homogeneity of variance was also assessed using Levene’s test. Bacterial taxa that were identified in < 10% of the faecal samples were removed from the taxa analysis. Linear discriminant analysis (LDA) effect size was used to detect differentially abundant taxa at the species level, between the control and CHF groups. Among those bacterial species with differential abundance, the effects of age, consuming human food, living with another dog, MCS, total daily diuretic dose, presence of CHF, subclassification of CHF, and duration of CHF, on the log transformed read counts, were further explored using a general linear model in a backward stepwise manner. Significance was set at *p* < 0.05.

## Supplementary information

Supplementary Information.
