# Supplementary material for: The gut microbiome in dogs with congestive heart failure: a pilot study
Source: Sci Rep. 2020 Aug 13;10:13777. doi: 10.1038/s41598-020-70826-0 (PMC7426839; doi:10.1038/s41598-020-70826-0)

The gut microbiome in dogs with congestive heart failure: A pilot study

Joonbum Seo[1]*, Linda Matthewman[2], Dong Xia[3], Jenny Wilshaw[1], Yu-Mei Chang[3],

David John Connolly[1]

Supplementary Tables

Supplementary Table S1. Demographic characteristics with further subclassification of congestive heart failure (CHF). The p value is from comparison of control, LCHF and RCHF groups. BiCHF was not included due to the small sample size. Differences seen on post-hoc analysis are annotated as a,b,c. *One dog in the left CHF (LCHF) group had a missing muscle condition score. **Urea and creatinine were only available for 10 dogs with LCHF, 11 dogs with right CHF (RCHF) and 2 dogs with biventricular CHF (BiCHF). ***The duration of CHF and diuretic dose were only compared between the LCHF and RCHF groups. BCS, body condition score, JRT, Jack Russell Terrier, James Wb, James Wellbeloved, Royal Canin AM, Royal Canin Adult Maintenance. The results are displayed as median [25th, 75th percentiles].

	Control (n=15)	LCHF (n=16)	RCHF (n=15)	BiCHF (n=4)	p value
Age (years)	8.0 [6.8-10.0]	10.8 [9.1-11.8][a]	8.8 [2.2-10.4][a]	10.4 [8.6-11.4]	**0.012**
Sex					0.501
Female (%)	7 (46.7%)	4 (25%)	6 (40%)	0	
Neutered:Entire	7:0	4:0	6:0	-	
Male (%)	8 (53.3%)	12 (75%)	9 (60%)	4 (100%)	
Neutered:Entire	5:3	9:3	2:7	4:0	
Body weight (kg)	20.3 [12.1-33.0][a]	7.9 [4.5-11.7][a,b]	42.6 [27.0-52.0][b]	25.5 [10.3-40.8]	**<0.001**
BCS (/9)	[a,b]	[a]	[b]		**0.007**
1/9	0	0	1 (6.7%)	0	
2/9	0	0	3 (20.0%)	1 (25.0%)	
3/9	0	3 (18.8%)	4 (26.7%)	1 (25.0%)	
4/9	3 (20.0%)	9 (56.3%)	3 (20.0%)	2 (50.0%)	
5/9	6 (40.0%)	3 (18.8%)	3 (20.0%)	0	
6/9	6 (40.0%)	1 (6.3%)	1 (6.7%)	0	
Muscle condition*	[a,b]	[a,c]	[b,c]		**<0.001**
Normal	15 (100%)	6 (37.5%)	2 (13.3%)	0	
Mild	0	7 (43.8%)	2 (13.3%)	2 (50%)	
Moderate	0	2 (12.5%)	7 (46.7%)	0	
Severe	0	6 (37.5%)	4 (26.7%)	2 (50%)	
Appetite	[a,b]	[a,c]	[b,c]		**<0.001**
Normal	15 (100%)	11 (68.8%)	2 (13.3%)	0	
Reduced	0	5 (31.3%)	13 (86.7%)	4 (100%)	
Urea (mmol/L)**	-	6.0 (±1.4)	6.0 (±4.9)	3.9 (±0.3)	0.950
Creatinine (µmol/L)**	-	97.2 (±26.5)	123.8 (±70.7)	106.1 (±26.5)	0.154
Duration of CHF (weeks)***	0	18.9 [4.3-34.0]	3 [2.0-8.6]	13.5 [4.5-45.8]	**0.036**
Medication					
Diuretic dose*** (mg/kg/day)	-	5.0 [3.5-6.9]	3.8 [2.6-4.4]	8.2 [4.8-9.9]	0.172
Furosemide	0	13 (81.3%)	12 (80.0%)	3 (75.0%)	-
Torsemide	0	3 (18.8%)	0	1 (25.0%)	-
Pimobendan	0	16 (100%)	13 (86.7%)	4 (100%)	-
Benazepril	0	12 (75.0%)	10 (66.7%)	3 (75.0%)	-
Spironolactone	0	12 (75.0%)	8 (53.3%)	3 (75.0%)	-
Fish Oil	0	0	2 (13.3%)	2 (50.0%)	-
Diltiazem/ Digoxin	0	1 (6.3%)	7 (46.7%)	1 (25.0%)	-

Breeds (%)	[a]	[a,b]	[b]		**<0.001**
-Small breeds	4 (26.7%)	14 (87.5%)	2 (13.3%)	2 (50.0%)	
Boston Terrier	0	0	1	0	
Brussell Griffin	0	1	0	0	
Chihuahua	0	3	0	0	
CKCS	0	6	0	2	
Cocker Spaniel	2	0	0	0	
Daschund	0	1	0	0	
Havanese	0	1	0	0	
JRT	1	0	1	0	
Pug	1	0	0	0	
Yorkshire Terrier	0	2	0	0	
-Large breeds	11 (73.3%)	2 (12.5%)	13 (86.7%)	2 (50.0%)	
Crossbred	4	1	2	1	
English Bulldog	1	0	1	0	
English Mastiff	0	0	0	0	
French Mastiff	0	0	4	0	
Golden Retriever	0	0	2	0	
Labradoodle	1	1	1	0	
Labrador	4	0	1	1	
Newfoundland	1	0	2	0	
**Dietary information**					
Human food	5 (33.3%)	11 (68.8%)	9 (60.0%)	3 (75.0%)	0.122
Dog food	15 (100%)	15 (93.8%)	14 (93.3%)	4 (100%)	0.602
Raw food	2 (13.3%)	1 (6.3%)	1 (6.7%)	1 (25.0%)	0.739
Main diet brand/type					0.405
Any dog food	3 (20.0%)	9 (56.3%)	5 (33.3%)	2 (50.0%)	
Arden Grange	1 (6.7%)	0	1 (6.7%)	0	
Barking Heads	0	0	1 (6.7%)	0	
Cesar	0	1 (6.3%)	0	0	
Canagan	1 (6.7%)	0	0	0	
Homecooked	0	1 (6.3%)	3 (20.0%)	1 (25.0%)	
James Wb	1 (6.7%)	2 (12.5%)	0	0	
Irish	0	0	0	1 (25.0%)	
Lily's Kitchen	1 (6.7%)	1 (6.3%)	0	0	
Purina Omega	1 (6.7%)	0	1 (6.7%)	0	
Royal Canin AM	1 (6.7%)	0	0	0	
Unknown brand	3 (20.0%)	2 (12.5%)	3 (20.0%)	0	
Tails.com	1 (6.7%)	0	0	0	
Wainwright's	2 (13.3%)	0	0	0	

Supplementary Table S2. Relative abundance % of bacterial species in control and congestive heart failure (CHF) groups. The results are displayed as median [25th, 75th percentiles]. Bacterial species (spp.) with clinically significant differential abundance are annotated using bold text.

Bacterial species	Control (n=15)	CHF (n=35)	p value
**Actinobacteria**			
Corynebacterium unclassified spp. 1	0 [0-0]	0 [0-0]	0.091
Corynebacterium unclassified spp. 2	0 [0-0]	0 [0-0]	0.127
Bifidobacterium unclassified spp.	0 [0-0]	0 [0-0]	0.988
Collinsella unclassified spp.	0 [0-0]	0 [0-0.02]	0.087
Collinsella stercoris	0.58 [0.17-1.63]	0.61 [0.17-1.41]	0.992
Slackia unclassified spp.	0.02 [0-0.05]	0.03 [0.01-0.07]	0.462
**Bacteroidetes**			
[Paraprevotellaceae] unclassified spp.	0 [0-0.11]	0 [0-0.01]	0.28
[Prevotella] unclassified spp.	1.49 [0.65-7.39]	0.31 [0.04-3.47]	0.07
Bacteroides unclassified spp.	0 [0-0.02]	0.01 [0-0.13]	0.093
Bacteroides unclassified spp.	8.13 [2.7-14.85]	7.54 [1.9-16.25]	0.874
Bacteroides fragilis	0 [0-0]	0 [0-0]	0.875
Bacteroides ovatus	0 [0-0]	0 [0-0.02]	0.099
Bacteroides plebeius	0.84 [0.14-2.11]	0.19 [0.02-1.09]	0.096
Bacteroides uniformis	**0 [0-0]**	**0 [0-0.05]**	**0.033**
Parabacteroides unclassified spp.	0 [0-0.01]	0 [0-0.05]	0.058
Parabacteroides distasonis	**0 [0-0]**	**0 [0-0.01]**	**0.038**
Prevotella copri	0.32 [0.02-6.93]	0.04 [0.03-3.66]	0.649
Bacteroidales S24-7 unclassified spp.	0 [0-0]	0 [0-0]	0.624
**Firmicutes**			
Staphylococcus unclassified spp.	0 [0-0]	0 [0-0]	0.127
***Enterococcaceae* unclassified spp.**	**0 [0-0.01]**	**0.02 [0-0.19]**	**0.01**
Lactobacillus unclassified spp.	0.02 [0.02-0.03]	0.03 [0.02-0.04]	0.07
Lactobacillus unclassified spp.	0 [0-0]	0 [0-0]	0.32
Leuconostoc unclassified spp.	0 [0-0.01]	0 [0-0.01]	0.584
Streptococcus unclassified spp.	0 [0-0]	0 [0-0]	0.212
Streptococcus luteciae	0.05 [0.02-0.09]	0.05 [0.02-0.21]	0.582
Turicibacter unclassified spp.	0.39 [0.09-1.01]	0.08 [0.03-1.16]	0.304
Clostridiales unclassified spp. 1	0 [0-0]	0 [0-0.04]	0.074
Clostridiales unclassified spp. 2	0.01 [0-0.09]	0.02 [0-0.22]	0.239
[Mogibacteriaceae] unclassified spp.	0 [0-0]	0 [0-0.02]	0.224
Clostridiaceae unclassified spp.	0 [0-0.05]	0 [0-0.06]	0.466
Clostridiaceae unclassified spp.	0 [0-0]	0 [0-0]	0.583
Clostridium unclassified spp.	0.03 [0-0.38]	0.04 [0-0.6]	0.836
Clostridium hiranonis	18.17 [7.68-27.1]	10.34 [3.82-17.75]	0.058
Clostridium perfringens	0.23 [0.09-0.75]	0.31 [0.1-4.69]	0.357
Sarcina unclassified spp.	0 [0-0.02]	0 [0-0.03]	0.841
Clostridiaceae SMB53 unclassified spp.	0.1 [0-0.45]	0.02 [0-0.77]	0.948
Lachnospiraceae unclassified spp.	0.81 [0.32-1.41]	0.71 [0.46-1.37]	0.634
Lachnospiraceae	1.25 [0.6-1.94]	0.86 [0.27-1.55]	0.315
[Ruminococcus] unclassified spp.	0.16 [0.06-0.42]	0.12 [0.06-0.48]	0.791
[Ruminococcus] gnavus	1.51 [0.52-2.82]	0.99 [0.5-1.7]	0.415
[Ruminococcus] torques	0.05 [0-0.17]	0 [0-0.11]	0.348
Blautia unclassified spp. 1	0.46 [0.35-1.7]	0.38 [0.09-0.93]	0.38
Blautia unclassified spp. 2	0.99 [0.59-2.57]	0.92 [0.23-1.84]	0.452
Blautia producta	1.9 [1.48-4.57]	1.85 [0.99-5.93]	0.546
***Clostridium* unclassified spp**.	**0.03 [0-0.11]**	**0 [0-0.02]**	**0.034**
Clostridium colinum	0 [0-0]	0 [0-0.03]	0.354
Coprococcus unclassified spp.	0 [0-0.03]	0 [0-0.04]	0.811
Dorea unclassified spp.	2.03 [1.16-2.37]	1.42 [0.66-4.02]	0.634
Epulopiscium unclassified spp.	0 [0-0]	0 [0-0.03]	0.206
***Roseburia* unclassified spp.**	**0 [0-0.04]**	**0.04 [0-0.14]**	**0.025**
Peptococcus unclassified spp.	0 [0-0.04]	0.01 [0-0.68]	0.16
Peptostreptococcaceae unclassified spp.	0.09 [0.02-0.46]	0.12 [0.01-0.97]	0.678
Peptostreptococcaceae unclassified spp.	0.31 [0.07-0.83]	0.33 [0.04-1.28]	0.975
[Clostridium] sordellii	0 [0-0.11]	0.02 [0-0.42]	0.316

Ruminococcaceae unclassified spp.	0.12 [0.03-0.28]	0.06 [0.01-0.12]	0.134
Ruminococcaceae unclassified spp.	0.27 [0.11-0.52]	0.19 [0.04-0.89]	0.832
Butyricicoccus pullicaecorum	0.06 [0.01-0.13]	0.04 [0.01-0.1]	0.544
Faecalibacterium prausnitzii	1.58 [0.23-3.31]	0.13 [0.04-2.78]	0.074
Oscillospira unclassified spp.	0.01 [0-0.05]	0.02 [0-0.12]	0.721
Ruminococcus unclassified spp.	0.01 [0-0.04]	0.04 [0.01-0.08]	0.168
Dialister unclassified spp.	0 [0-0]	0 [0-0]	0.179
Megamonas unclassified spp.	0.54 [0.12-2.76]	0.39 [0.06-2.03]	0.518
Phascolarctobacterium unclassified spp.	0.13 [0.01-0.59]	0.19 [0-0.52]	0.949
Erysipelotrichaceae unclassified spp.	0 [0-0.02]	0 [0-0]	0.194
***Erysipelotrichaceae* unclassified spp.**	**0.21 [0.07-0.55]**	**0.01 [0-0.11]**	**0.008**
[Eubacterium] biforme	**0.21 [0.01-1.06]**	**0.01 [0.01-0.16]**	**0.028**
[Eubacterium] dolichum	0.05 [0.01-0.09]	0.03 [0-0.14]	0.823
***Allobaculum* unclassified spp.**	**0.69 [0.1-1.43]**	**0.27 [0.03-0.8]**	**0.038**
***Catenibacterium* unclassified spp.**	**0.14 [0.05-3.3]**	**0.03 [0.02-0.21]**	**0.032**
Clostridium cocleatum	**0.1 [0.02-0.17]**	**0.04 [0-0.11]**	**0.028**
Clostridium saccharogumia	0 [0-0]	0 [0-0.01]	0.105
Coprobacillus unclassified spp.	0 [0-0.04]	0 [0-0.05]	0.898
**Fusobacteria**			
Fusobacteriaceae unclassified spp.	1.3 [0.4-3.92]	0.83 [0-2.2]	0.257
Fusobacteriaceae unclassified spp.	16.49 [12.48-29.33]	11.29 [6.21-20.09]	0.097
Cetobacterium somerae	0 [0-0]	0 [0-0.01]	0.224
Fusobacterium unclassified spp.	4.3 [2.5-8.02]	3.57 [1.1-10.36]	0.604
**Proteobacteria**			
Sutterella unclassified spp.	1.33 [0.36-2.99]	1.11 [0.31-2.53]	0.664
Bilophila unclassified spp.	0 [0-0]	0 [0-0]	0.091
Desulfovibrio unclassified spp.	0 [0-0]	0 [0-0]	0.091
Campylobacter unclassified spp.	0 [0-0.09]	0 [0-0.05]	0.709
Helicobacteraceae unclassified spp.	0 [0-0]	0 [0-0.05]	0.156
Flexispira unclassified spp.	0 [0-0.15]	0 [0-0.03]	0.214
Helicobacter unclassified spp.	0 [0-0.04]	0 [0-0]	0.404
Aeromonadaceae unclassified spp.	0 [0-0]	0 [0-0]	0.339
Succinivibrionaceae unclassified spp.	0.03 [0-0.31]	0.02 [0-0.08]	0.79
Anaerobiospirillum unclassified spp.	0.01 [0-0.46]	0.01 [0-0.44]	0.886
Succinivibrio unclassified spp.	0 [0-0]	0 [0-0]	0.127
***Enterobacteriaceae* unclassified spp.**	**0.07 [0.04-0.22]**	**0.3 [0.11-3.49]**	**0.002**
Escherichia coli	**0 [0-0]**	**0 [0-0.01]**	**0.033**

Supplementary Table S3. Taxa analysis at the Phylum level. The median percentage abundance [IQR] is displayed for the control, left congestive heart failure (LCHF), right congestive heart failure (RCHF) and biventricular congestive heart failure (BiCHF) groups. BiCHF group was not included in the statistical analysis due to the small number. The results are displayed as median [25th, 75th percentiles].

Phylum	Control (n=15)	LCHF (n=16)	RCHF (n=15)	BiCHF (n=4)	p value
Firmicutes (%)	46.8 [36.7-57.0]	51.5 [32.9-61.7]	42.2 [31.1-58.8]	63.2 [32.6-72.0]	0.608
Fusobacteria (%)	25.8 [19.6-36.6]	20.5 [11.5-29.6]	23.1 [12.3-36.8]	11.2 [2.6-45.2]	0.313
Bacteroides (%)	17.7 [10.1-25.2]	23.9 [6.8-29.7]	15.9 [2.5-27.6]	17.9 [4.3-21.8]	0.919
Proteobacteria (%)	2.6 [1.0-4.9]	7.0 [1.6-13.0]	6.7 [2.4-8.5]	5.4 [3.0-18.0]	0.104
Actinobacteria (%)	0.6 [0.2-1.7]	0.8 [0.2-2.6]	0.8 [0.6-1.5]	0.9 [0.2-1.8]	0.963

Supplementary Figures

Supplementary Figure S1. Comparison of alpha (A, C and E) and beta diversity (B, D and F) between the control vs congestive heart failure (CHF) groups (A and B); control vs CHF dogs living alone vs CHF dogs living with other dogs (C and D); and control living with CHF dogs vs CHF dogs living with healthy control dogs (E and F). The remaining dogs in the graphs E and F are still displayed for visual interpretation but not included in the statistical analysis. The line in the alpha rarefaction curves marks the mean and the error bars mark the standard deviation. The principle coordinate analysis plot shows the results of Bray-Curtis dissimilarity analysis.

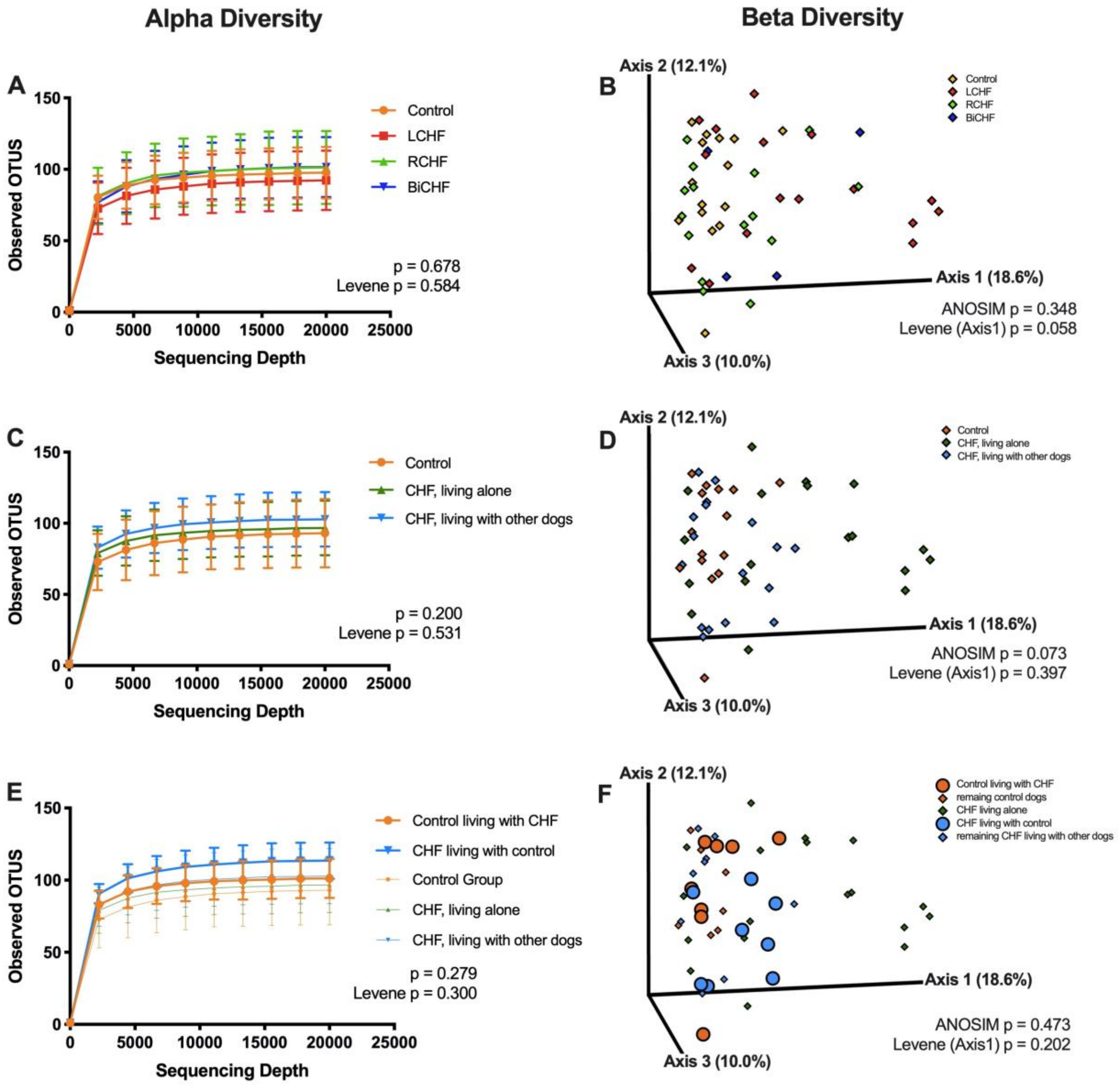

Supplement: Supplementary file 1 — Supplementary Information. [file 41598_2020_70826_MOESM1_ESM.pdf]
